# The Interaction of the Transmembrane Domain of SARS-CoV-2 E-Protein with Glycyrrhizic Acid in Lipid Bilayer

**DOI:** 10.3390/membranes13050505

**Published:** 2023-05-10

**Authors:** Polina A. Kononova, Olga Yu. Selyutina, Nikolay E. Polyakov

**Affiliations:** Voevodsky Institute of Chemical Kinetics and Combustion, Institutskaya str. 3, 630090 Novosibirsk, Russia

**Keywords:** glycyrrhizic acid, antiviral activity, SARS-CoV-2, coronavirus E-protein, lipid membranes, NMR, NOESY

## Abstract

The interaction of the transmembrane domain of SARS-CoV-2 E-protein with glycyrrhizic acid in a model lipid bilayer (small isotropic bicelles) is demonstrated using various NMR techniques. Glycyrrhizic acid (GA) is the main active component of licorice root, and it shows antiviral activity against various enveloped viruses, including coronavirus. It is suggested that GA can influence the stage of fusion between the viral particle and the host cell by incorporating into the membrane. Using NMR spectroscopy, it was shown that the GA molecule penetrates into the lipid bilayer in a protonated state, but localizes on the bilayer surface in a deprotonated state. The transmembrane domain of SARS-CoV-2 E-protein facilitates deeper GA penetration into the hydrophobic region of bicelles at both acidic and neutral pH and promotes the self-association of GA at neutral pH. Phenylalanine residues of the E-protein interact with GA molecules inside the lipid bilayer at neutral pH. Furthermore, GA influences the mobility of the transmembrane domain of SARS-CoV-2 E-protein in the bilayer. These data provide deeper insight into the molecular mechanism of antiviral activity of glycyrrhizic acid.

## 1. Introduction

**Figure 1:**
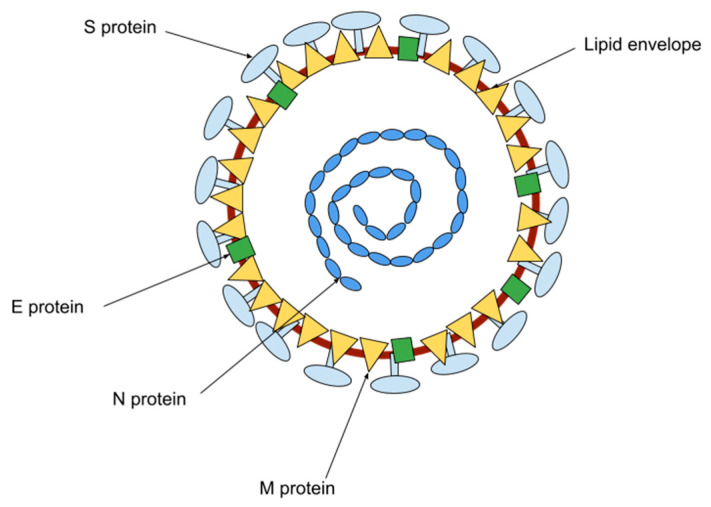
Schematic representation of the coronavirus structure.

The coronavirus particles contain RNA tightly packed into the center of the particle, surrounded by a protective capsid. The nucleocapsid protein (N) forms the capsid outside the genome, and the genome is further packed by a lipid envelope which is associated with three structural proteins: membrane protein (M), spike protein (S), and envelope protein (E) (Figure 1) [1].

E-protein of SARS-CoV-2 is composed of 75 amino acids. It consists of three domains: a transmembrane domain (ETM) (residues 17–37), an intermediate helical domain, and N- and C-terminal domains. The ETM of SARS-CoV-2 is highly hydrophobic [2]. This protein is a minor virion structural component that plays an important, not fully understood role in virus production. It is an integral membrane protein involved in several aspects of the virus’s life cycle, such as assembly, budding, envelope formation, and pathogenesis. Structurally, the E proteins are conserved across the different coronavirid groups but have little sequence similarity between the groups [3]. Thus, due to high conservativity, E-protein could be a promising target for antiviral agents. It was shown for different coronaviruses that E-protein, together with membrane glycoprotein (M-protein), is necessary for the production of virus-like particles. It was shown that neither the nucleocapsid protein nor the spike protein is necessary for viral budding, and it was shown that this is a general phenomenon of the coronaviruses [4,5,6].

During the replication cycle, E-protein is abundantly expressed inside the infected cell, but only a small portion is incorporated into the virus envelope. The majority of the protein participates in viral assembly and budding [7,8]. It can act as a viroporin by oligomerizing after insertion in host membranes to create a hydrophilic pore that allows ion transport [9,10]. The assembly of virions is thought to take place at intracellular membranes in the region of the endoplasmic reticulum Golgi complex (ERGIC). The deletion of the gene of SARS-CoV E protein results in virus yields that are 20- to 200-fold lower than those of the wildtype virus, depending on the cell type [11].

E-protein forms a homopentameric cation channel in the lipid membrane that is important for virus pathogenicity. ETM forms a five-helix bundle surrounding a narrow pore [12]. The protein deviates from the ideal α-helical geometry due to three phenylalanine residues, which stack within each helix and between helices. Hexamethylene amiloride binds the polar amino-terminal lumen, whereas acidic pH affects the carboxy-terminal conformation. Thus, the N- and C-terminal halves of this bipartite channel may interact with other viral and host proteins semi-independently. The structure sets the stage for designing E inhibitors as antiviral drugs [12]. A study on the role of E protein and pores formed by ETM in viral release is lacking [13]. However, on the basis of the studies of the role of influenza virus viroporin, it is suggested that it can induce membrane scission and virion budding by altering membrane cholesterol [13].

**Figure 2:**
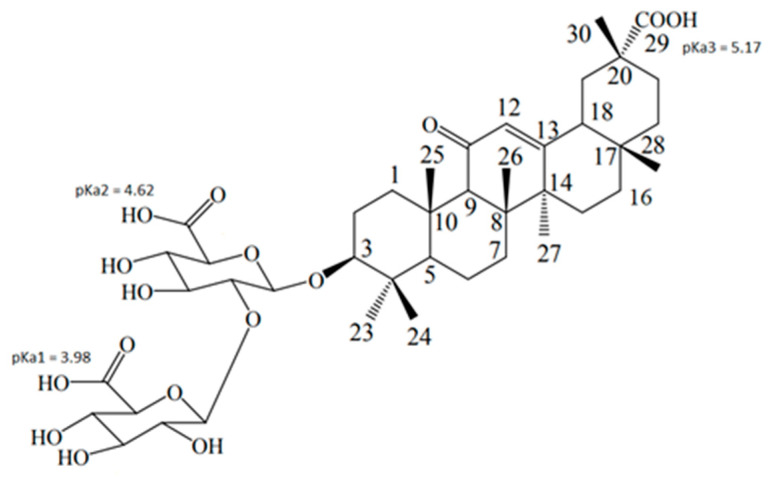
Structure of glycyrrhizic acid.

Glycyrrhizic acid (GA, Figure 2) is the active component of licorice root, which exhibits a wide range of biological activities, including anti-inflammatory and antiviral activities [14,15,16]. In particular, a virus-inhibiting effect of GA on SARS-associated coronavirus was demonstrated [17]. Recently, a number of studies indicated the promise of using GA for the treatment of SARS-CoV-2 [18,19]. In addition, there are data on the activity of GA derivatives, mono-, di-, and trinicotinates, against the SARS-CoV-2 virus in vitro [20]. Despite the abundance of studies on the activity of GA against various DNA and RNA viruses, the mechanism of its antiviral action remains unclear. It has been shown that the effect of GA on the herpes virus associated with Kaposi’s sarcoma is associated with the inhibition of viral RNA synthesis [21]. In a number of studies, the effect of GA on the replication of various viruses was found [22,23]. However, the fact that the antiviral activity of GA is found in relation to various weakly interconnected DNA and RNA viruses suggests that this effect is not only associated with the effect on RNA synthesis.

It was found that, when GA is added 6 h after cell infection with the Epstein–Barr virus, no antiviral effect is observed [24]. At the same time, when GA is added immediately after infection and the cells are subsequently washed after 5 h, the antiviral effect remains irreversible. Accordingly, it is concluded that GA selectively blocks the penetration of the virus into the cell, since this process occurs approximately in the first 5 h after infection. In the case of porcine respiratory syndrome virus, it was shown that the action of GA is mainly associated with the stage of virus penetration and has little effect on the stages of absorption and release of the virus [25]. A number of studies have shown that GA and its derivatives prevent the penetration of a number of viruses through the plasma membrane [17,24,26,27,28]. In addition, it was found that the action of GA leads to a decrease in the fluidity of cell membranes [26,29]. It was also found that GA is able to inhibit the release of viral particles from an infected cell [30]. Previously, it was shown that GA could penetrate into the model lipid membrane, affecting its properties (lipid mobility, phase transition temperature, membrane elasticity, and membrane permeability) [31,32,33,34,35]. Thus, one of the possible mechanisms of the antiviral action of GA is to prevent the fusion of the lipid envelope of the virus with the plasma membrane of the host cell. In addition, molecular docking shows the possibility of embedding glycyrrhizic acid into the pore formed by the E-protein pentamer [36]. We believe that the membrane-modifying ability of GA can also indirectly affect the activity of the E-protein built into the lipid envelope of the virus. Therefore, due to the unclear role of the E-protein in the functioning and pathogenesis of coronaviruses in general and SARS-CoV-2 in particular, comprehensive studies are required on both the interaction of the protein itself with the lipid membrane and the effect of various inhibitors on these interactions and on E-protein activity.

In the present work we studied the interaction of GA with the transmembrane domain of SARS-CoV-2 E-protein in a model lipid bilayer. Although the transmembrane domain is only a fragment of the E-protein, it is considered as a target for various inhibitors, and the use of a transmembrane domain embedded in the lipid surrounding is a good model for drug–target interaction [12,37]. Small isotropic bicelles (bilayer micelles) were used as a model of a lipid bilayer. Lipid bicelles are a widely used membrane model in solution ^1^H-NMR studies [38]. They are free from the main drawback of detergent micelles associated with the high curvature of the surface of micelles. Bicelles have a bilayer structure, which closely corresponds to the organization of the lipid membrane, but they are still relatively small in size, which allows analysis by solution ^1^H-NMR. The bicelle size, determined by the DMPC/DHPC concentration ratio (q), plays an important role in peptide distribution in the lipid bilayer. It was found that significant changes in peptide distribution occur when the q value is below 0.5 [39]. It is supposed that q values between 0.5 and 0.6 are optimal for the characterization of protein position in the lipid bilayer by means of solution NMR [39].

## 2. Materials and Methods

### 2.1. Materials

GA (purity >98%, Shaanxi Pioneer Biotech Co., Ltd., Xi’an, China) and the transmembrane domain of SARS-CoV-2 E-protein (ETGTLIVNSVLLFLAFVVFLLVTLAILTALR, purity >96%, Pepmic, Suzhou, China) were used as received.

**Figure 3:**
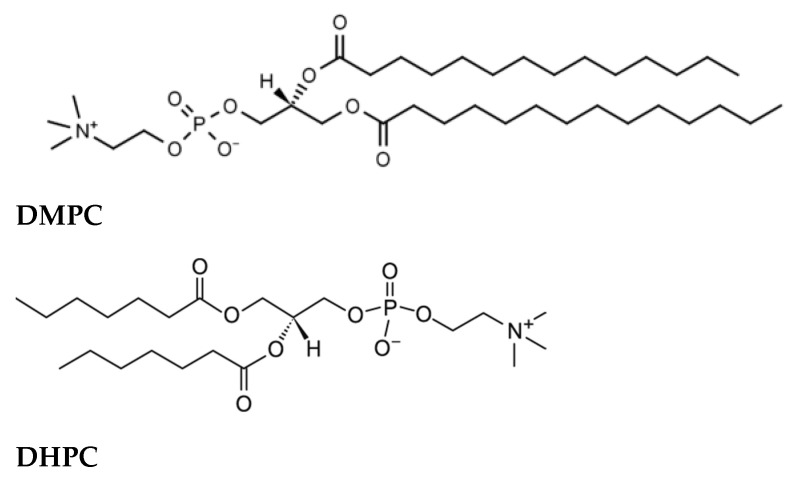
Structures of the phospholipids 1,2-dimyristoyl-*sn*-glycero-3-phosphocholine (DMPC) and 1,2-diheptanoyl-*sn*-glycero-3-phosphocholine (DHPC).

Bicelles were formed from DMPC (1,2-dimyristoyl-*sn*-glycero-3-phosphocholine) and DHPC (1,2-diheptanoyl-*sn*-glycero-3-phosphocholine, purity > 99%; Avanti Polar Lipids, Alabaster, AL, USA, Figure 3). 

Powdered components (lipids, ETM) were dissolved in ethanol, the solvent was dried, and the resulting film was hydrated with D_2_O (99.9% D, Cambridge Isotope Laboratories, Tewksbury, MA, USA). The DMPC/DHPC molar ratio was 1:2, with the total lipid concentration being 12 mM. Phosphate-buffered saline (PBS) was used to obtain a pH = 7.4. DCl was added to enable the solution to achieve pH = 3. To form bicelles, three freeze–thaw cycles were performed.

### 2.2. NMR Study

^1^H-NMR and selective NOESY (nuclear Overhauser effect spectroscopy) spectra were recorded in D_2_O on a Bruker Avance HD III NMR spectrometer (500 MHz ^1^H operating frequency). T_1_ relaxation times were measured using a standard inversion-recovery pulse sequence. NMR spectra were processed in the TopSpin 4.1.4 software. 

The mixing time in selective NOESY was determined by a series of selective gradient NOESY experiments (sNOESY). The optimal mixing time, i.e., the delay during which the magnetization transfer via cross-relaxation occurs, was chosen to be 0.6 s. During the measurements, the temperature was kept at 300 K.

## 3. Results and Discussion

### 3.1. GA Interaction with Lipid Bilayer

The ^1^H-NMR NOESY technique allows determining the localization and distribution of molecules in membranes. NOESY cross-peaks are observed at distances less than 0.5 nm. In the present study, we applied this technique to study the interaction of GA and ETM with bicelles. 

**Figure 4:**
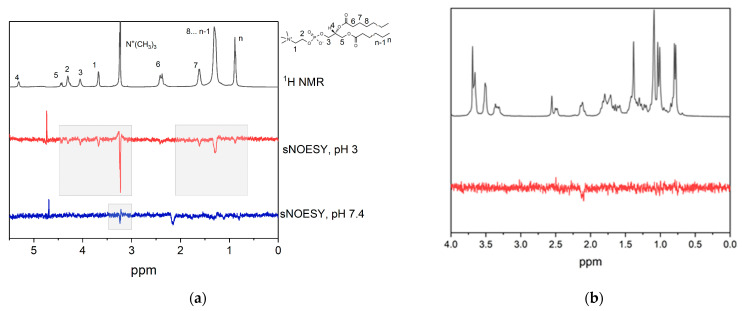
(**a**) The fragments of ^1^H-NMR and selective NOESY (sNOESY) spectra of 1 mM GA in DMPC/DHPC bicelles (total lipid concentration 12 mM) in D_2_O at pH = 3 and pH = 7.4. Selective excitation of the GA signal at 5.6 ppm (proton 12 from Figure 1) was performed. (**b**) 2 mM GA in water, pH 7.4. Selective excitation of GA proton (12) was performed.

Figure 4 shows the fragments of ^1^H-NMR and selective gradient NOESY (sNOESY) spectra of GA in DMPC/DHPC bicelles in D_2_O with different pH values. The pKa values of GA in solution are 3.98, 4.62, and 5.17 (Figure 2) [40]. In the sample with pH = 3, cross-peaks of GA proton (12) with all lipid signals were observed, whereas, in the sample with pH = 7.4, only the cross-peak with N^+^(CH_3_)_3_ was observed. The signal at 2.1 ppm corresponds to GA proton (18), which is close to GA proton (12) due to the features of the chemical structure. The same cross-peak was observed for GA solution in water without lipids (Figure 4b). The obtained results correlate with the observed earlier results of a molecular dynamics simulation of protonated and deprotonated GA interaction with a lipid bilayer [33,34,35]. The simulation results revealed that, in a fully deprotonated state, GA is located on the surface of bilayer, whereas, when it is fully protonated, it is located inside the bilayer. This led to the assumption that, at pH = 3, GA is protonated, whereas, at pH = 7.4, GA is deprotonated.

### 3.2. GA Interaction with ETM in Bicelles

**Figure 5:**
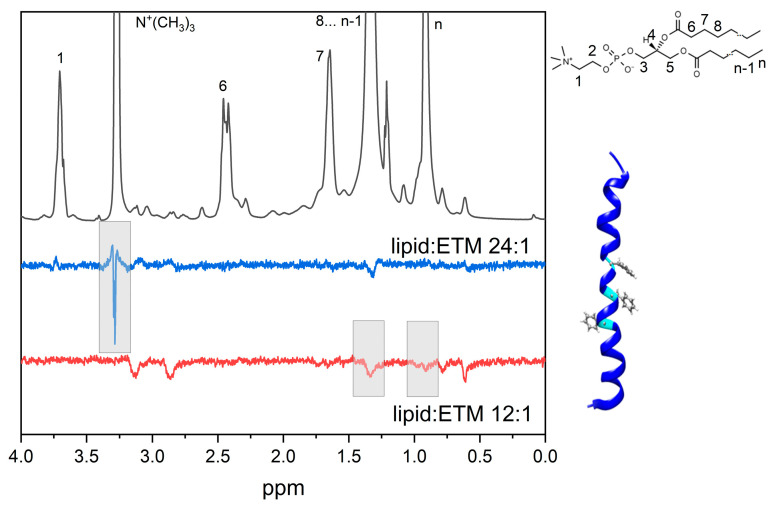
Fragments of ^1^H-NMR (black line) and selective NOESY spectra of ETM in DMPC/DHPC bicelles (lipid/ETM ratio 12:1, red line; lipid/ETM ratio 24:1, blue line), pH 7.4. Selective excitation of ETM phenylalanine protons was performed. Phenylalanine residues are shown in ball-and-stick representation.

Figure 5 shows the ^1^H-NMR and 1D selective NOESY spectra of DMPC/DHPC bicelles containing ETM at different lipid/peptide ratios. The protons of phenylalanine (7.3–7.5 ppm) were selectively excited. A cross-peak between phenylalanine protons and N^+^(CH_3_)_3_ at a lipid/peptide ratio of 24:1 indicates that the phenylalanine residue is located near the bilayer surface. A disappearance of this cross-peak with the increase in ETM concentration with respect to lipid, along with an appearance of a cross-peak between phenylalanine protons and CH_3_ groups of lipids, indicates that the phenylalanine residue is located inside the bilayer. Furthermore, cross-peaks between phenylalanine protons and other ETM residues can be observed. The same results were obtained for pH = 3 and pH = 7.4. The literature data state that, in DPC micelles with a lipid/peptide ratio of 100:1, SARS ETM is in pentameric form [37]. According to the literature, DPC micelles contain 50–100 molecules [41], and a DPC/peptide ratio of 100:1 corresponds to approximately 1–2 peptide molecules per micelle. Taking the area per lipid for DMPC molecule as 65 Å [42] and the bicelle radius as 4 nm [43], it could be estimated that the DMPC/DHPC bicelle contains ~20 molecules per bicelle. Thus, lipid/peptide ratios of 12:1 and 6:1 correspond to approximately 1.7–3.3 peptide molecules per bicelle, which is close to the literature value at which ETM is stated to be in oligomeric form; however, a lipid/peptide ratio of 24:1 corresponds to approximately 0.65 ETM molecules per bicelle. It is known that pore-forming peptides in monomeric form are oriented parallel to bilayer surface, while pores formed by peptide oligomers are oriented perpendicular to the bilayer surface [44]. Thus, since phenylalanine residues are placed in the middle of peptide, their localization near the bilayer surface may be due to the presence of ETM in monomeric form.

**Figure 6:**
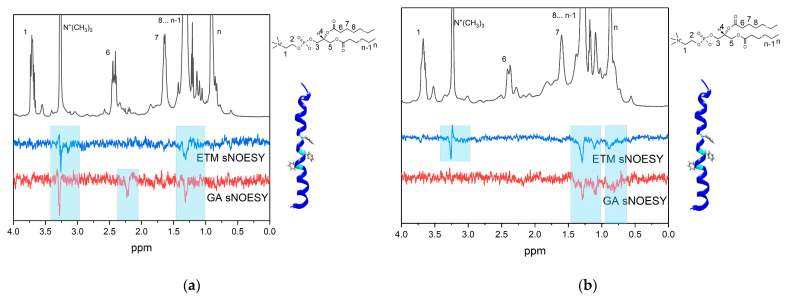
Fragments of ^1^H-NMR and selective NOESY spectra of (**a**) 2 mM GA and 1 mM ETM in DMPC/DHPC bicelles (total lipid concentration 12 mM), (**b**) 2 mM GA and 2 mM ETM in DMPC/DHPC bicelles.

Figure 6 shows the ^1^H-NMR and 1D selective NOESY spectra of DMPC/DHPC bicelles containing 2 mM GA and (A) 1 mM ETM or (B) 2 mM ETM at pH = 7.4. The proton (12) (5.6 ppm, Figure 1) of GA was selectively excited. In the case of ETM, the protons of phenylalanine (7.3–7.5 ppm) were selectively excited. 

In the case of pH = 7.4 and an ETM concentration of 1 mM, cross-peaks were observed between proton (12) of GA and the N^+^(CH_3_)_3_ and CH_2_ groups of lipids (Figure 6a). Thus, the GA molecule in a deprotonated state in the presence of a small ETM concentration was located inside the lipid bilayer. This differs significantly with the case of the absence of ETM molecules, whereby GA was located on the bilayer surface at pH = 7.4. The presence of ETM molecules probably facilitated the penetration of GA molecules into the membrane or influenced the pKa value of GA. A cross-peak between GA protons (12) and (18) (2.2 ppm) was also observed, replicating the observations for GA in bicelles and in D_2_O in the absence of ETM (Figure 4b). For ETM molecules, cross-peaks were observed between phenylalanine protons and the N^+^(CH_3_)_3_ and CH_2_ groups of lipids (Figure 6a). It is noticeable that a cross-peak between phenylalanine protons and N^+^(CH_3_)_3_ groups appeared, which was not observed in the absence of GA. Thus, GA influenced the localization of ETM inside the lipid bilayer.

With an increase in the ETM concentration (Figure 6b), the cross-peak between proton (12) of GA and the N^+^(CH_3_)_3_ group of lipids disappeared, but the cross-peak between proton (12) of GA and the CH_2_ group of lipids was still observed. In addition, a cross-peak between proton (12) of GA and the CH_3_ groups (0.9 ppm) of lipids was observed. This means that GA molecules penetrated more deeply into the lipid bilayer. For ETM molecules, cross-peaks were observed between phenylalanine protons and the N^+^(CH_3_)_3_ and CH_2_ groups of lipids, as seen at small ETM concentrations, but the cross-peak between phenylalanine protons and the CH_3_ group (0.9 ppm) of lipids was additionally observed. Comparing this result with that for ETM in the bilayer in the absence of GA, we can infer that some ETM molecules are in monomeric form, whereas others are in oligomeric form at high ETM concentrations in the presence of GA.

**Figure 7:**
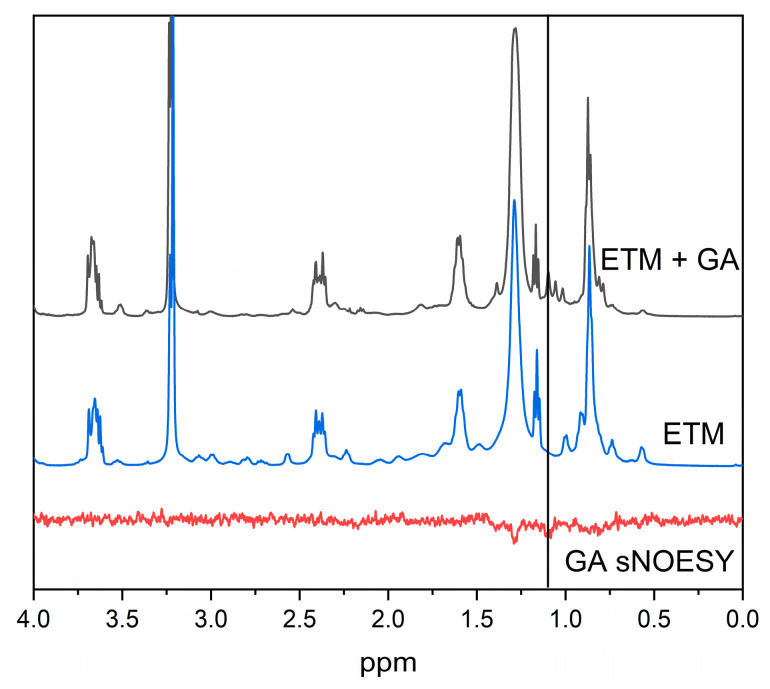
Fragments of ^1^H-NMR of 2 mM GA and 1 mM ETM in DMPC/DHPC bicelles (black), and 2 mM ETM in DMPC/DHPC bicelles (blue), as well as selective NOESY spectra of 2 mM GA and 2 mM ETM in DMPC/DHPC bicelles (red), pH = 7.4. Selective excitation of GA proton (12) and ETM phenylalanine protons was performed. Total lipid concentration = 12 mM.

It is noticeable that, both for proton (12) of GA and for phenylalanine protons of ETM, cross-peaks with the signal at 1.1 ppm were observed. A comparison of the ^1^H-NMR spectra of the samples containing 2 mM ETM and 2 mM ETM + 1 mM GA in bicelles (Figure 7) demonstrates that this signal corresponded to GA. According to [45], the GA signal at 1.1 ppm corresponds to proton (16). The presence of a cross-peak between protons (12) and (16) of GA, which are separated by a distance of more than 0.5 nm within a single molecule, indicates the presence of self-association. Thus, a high concentration of ETM promotes self-association of GA inside the lipid bilayer at neutral pH. The presence of a cross-peak between phenylalanine protons of ETM and (16) proton of GA indicates that GA molecules were located near phenylalanine residues in the lipid bilayer.

**Figure 8:**
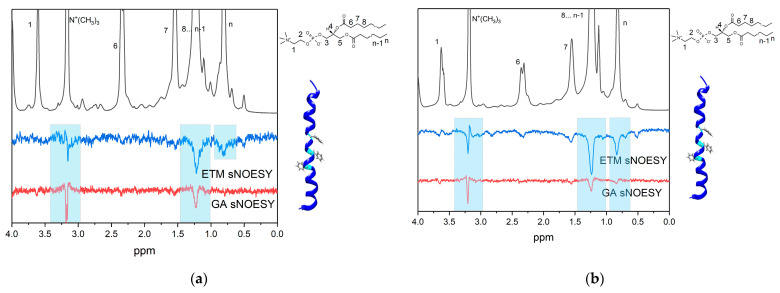
Fragments of ^1^H-NMR and selective NOESY spectra of (**a**) 2 mM GA and 1 mM ETM in DMPC/DHPC bicelles, and (**b**) 2 mM GA and 2 mM ETM in DMPC/DHPC bicelles, pH = 3. Selective excitation of GA proton (12) and ETM phenylalanine protons was performed. Phenylalanine residues are shown in ball-and-stick representation. Total lipid concentration = 12 mM.

Figure 8 shows the ^1^H-NMR and 1D selective NOESY spectra of DMPC/DHPC bicelles containing 2 mM GA and (A) 1 mM ETM or (B) 2 mM ETM at pH = 3. The proton (12) (5.6 ppm, Figure 1) of GA was selectively excited. In the case of ETM, the protons of phenylalanine (7.3–7.5 ppm) were selectively excited.

In the case of pH = 3 and an ETM concentration of 1 mM, cross-peaks were observed between proton (12) of GA and the N^+^(CH_3_)_3_ and CH_2_ groups of lipids (Figure 8a). Thus, the GA molecule in the presence of a small ETM concentration was located inside the lipid bilayer at acidic pH. It is noticeable that, at pH = 7.4, a cross-peak between phenylalanine protons and the N^+^(CH_3_)_3_ group appeared, which was not observed in the absence of GA.

With an increase in the ETM concentration (Figure 8b), an additional cross-peak between proton (12) of GA and the CH_3_ group (0.9 ppm) of lipids was observed. This means that GA molecules penetrated more deeply into the lipid bilayer, as seen at pH = 7.4. For ETM molecules, cross-peaks were observed between phenylalanine protons and the N^+^(CH_3_)_3_, CH_2_, and CH_3_ groups of lipids at both ETM concentrations. Comparing this result with the result for ETM in bilayer in the absence of GA, we can infer that some ETM molecules were in monomeric form, while others were in oligomeric form in the presence of GA. In contrast to pH = 7.4, no cross-peaks within GA and between ETM protons and proton (16) of GA were observed. This means that self-association of GA and association of GA with ETM did not take place at pH = 3.

**Figure 9:**
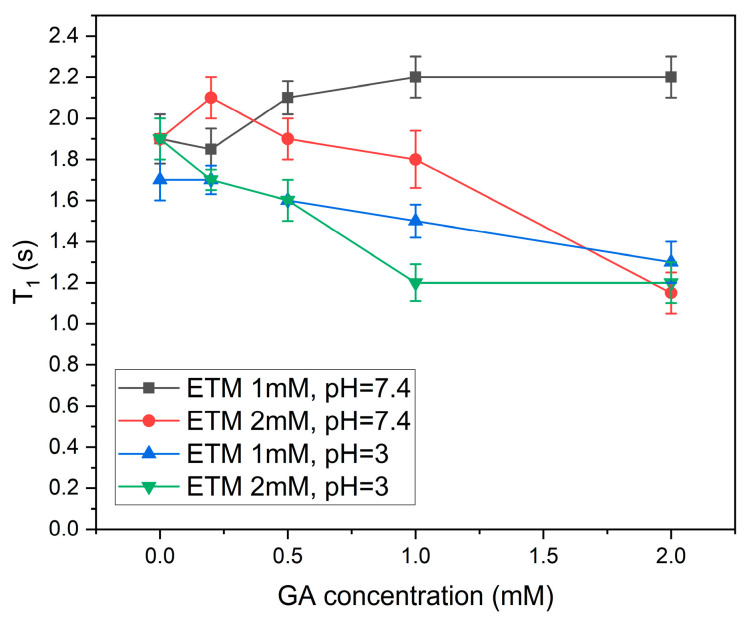
The dependence of spin–lattice (T1) relaxation times of ETM phenylalanine protons on GA concentration for samples with different ETM concentrations and pH values.

In addition, the dependence of spin–lattice (T_1_) relaxation times of ETM phenylalanine protons on GA concentration was measured (Figure 9). Spin–lattice relaxation times T_1_ are very sensitive to molecular mobility and intermolecular interactions. The relaxation times of the peptide decreased significantly for all samples except for that containing a small (1 mM) concentration of ETM with pH = 7.4. Taking into account that the specific interaction of phenylalanine protons with GA was proven only for the sample containing 2 mM ETM with pH = 7.4, it can be concluded that the decrease in mobility of ETM protons was caused by the influence of GA on lipid bilayer properties [31,35]. The absence of an effect in the case of the ETM concentration of 1 mM at pH = 7.4 may have been caused by shallow penetration of GA in this case.

We can conclude that, at pH = 7.4, GA molecules directly interacted with ETM phenylalanine residues inside the lipid bilayer, whereas, at pH = 3, GA molecules had an indirect lipid-mediated effect on SARS-CoV-2 E-protein.

## 4. Conclusions

It was found that, at pH = 3, GA was located inside the lipid bilayer, whereas, at pH = 7.4, GA was located on the bilayer surface. This correlates with previous molecular dynamic simulations, where GA was located in the middle of the bilayer in protonated form, but on the bilayer surface in deprotonated form. Taking into account the pKa values of GA in solution (pKa = 3.98, 4.62, and 5.17 [40]), we suggest that, in the absence of ETM in bicelles, GA is protonated at pH = 3 but deprotonated at pH = 7.4.

The selective NOESY showed the influence of the transmembrane domain of the coronavirus E-protein on the localization of GA in the lipid bilayer. Small isotropic DMPC/DHPC bicelles were used as a model of lipid bilayer. The presence of the transmembrane domain of coronavirus facilitates the deep penetration of GA in the center of the bilayer at both acidic and neutral pH. Furthermore, ETM promotes self-association of GA molecules, and ETM phenylalanine residues interact with GA inside the lipid bilayer at neutral pH. GA, in turn, influences the localization of ETM inside the lipid bilayer. Moreover, GA influences the mobility of ETM protons inside the bilayer. The changes in transmembrane domain mobility can influence the activity of the E-protein. 

**Figure 10:**
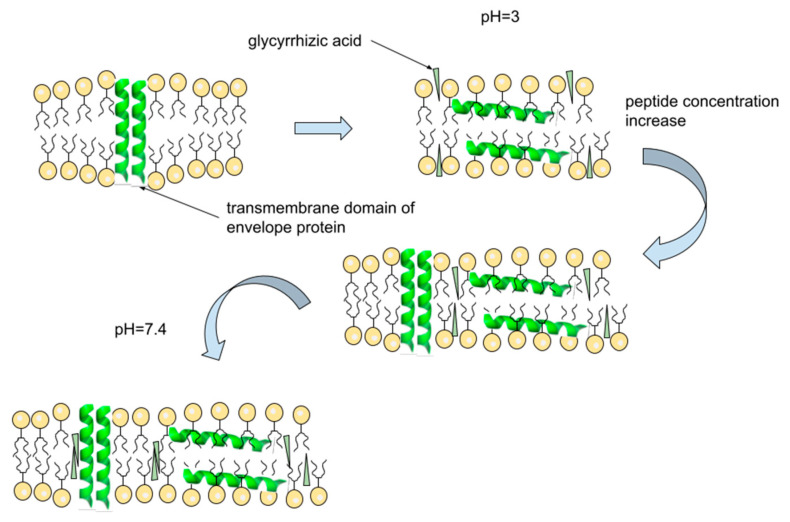
Schematic representation of proposed mechanism of GA interaction with ETM. The addition of GA leads to a partial violation on ETM oligomers, and GA molecules penetrate into the middle of the lipid bilayer near the pore formed by the ETM oligomer.

It was found that, in all cases, the mutual influence of GA and ETM on their localization took place, while a direct interaction of GA and ETM was observed only for a high concentration of ETM at pH = 7.4. Thus, the presence of GA leads to the displacement of phenylalanine protons from the center of the bilayer to its surface. Probably, the effect of GA on ETM localization is caused by the changes in lipid mobility in the presence of GA [34,35]. It is known that pore-forming peptides in monomeric form are oriented parallel to the bilayer surface, while pores formed by peptide oligomers are oriented perpendicular to the bilayer surface [44]. Thus, displacement of phenylalanine residues, located in the middle of peptide, from the bilayer center to the bilayer surface may be due to a violation of ETM self-association. A schematic representation of the proposed mechanism of GA interaction with ETM in bilayer is summarized in Figure 10.

It is known that pKa values of drugs and proteins can be significantly changed in a lipid environment [46,47]. pKa values can also be affected by hydrogen bonding [48]. Therefore, pKa values of GA can be changed in a lipid environment and in the presence of ETM molecules, and the observed changes in GA behavior may have also been caused by pKa changes. However, the observed differences in GA behavior at pH = 3 and pH = 7.4 in the presence of ETM molecules led to the suggestion that pKa changes may not be the only reason for GA localization changes in the presence of ETM. 

Previously, it was found that GA could significantly influence the mobility of lipids in a model bilayer [34,35], as well as cell membrane permeability and elasticity [31]. The obtained results revealed that the interaction of GA with the lipid bilayer strongly depended on the GA protonation state. All mentioned effects may have been influenced by the lipid composition of the model membrane. Further studies on the influence of lipid composition close to the endoplasmic reticulum/Golgi intermediate compartment on the interaction of GA and ETM are needed. We hope that the obtained results will stimulate further in vivo and in vitro studies of the transmembrane domain of coronavirus E-protein as a target for antiviral drugs.

## Data Availability

Data are provided in the article.

## References

[1] Brian D.A., Baric R.S. (2005). Coronavirus Genome Structure and Replication. Curr. Top. Microbiol. Immunol..

[2] Cao Y., Yang R., Lee I., Zhang W., Sun J., Wang W., Meng X. (2021). Characterization of the SARS-CoV-2 E Protein: Sequence, Structure, Viroporin, and Inhibitors. Protein Sci..

[3] Arbely E., Khattari Z., Brotons G., Akkawi M., Salditt T., Arkin I.T. (2004). A Highly Unusual Palindromic Transmembrane Helical Hairpin Formed by SARS Coronavirus E Protein. J. Mol. Biol..

[4] Baudoux P., Carrat C., Besnardeau L., Charley B., Laude H. (1998). Coronavirus Pseudoparticles Formed with Recombinant M and E Proteins Induce Alpha Interferon Synthesis by Leukocytes. J. Virol..

[5] Vennema H., Godeke G.J., Rossen J.W.A., Voorhout W.F., Horzinek M.C., Opstelten D.J.E., Rottier P.J.M. (1996). Nucleocapsid-Independent Assembly of Coronavirus-like Particles by Co-Expression of Viral Envelope Protein Genes. EMBO J..

[6] Bos E.C.W., Luytjes W., Van Der Meulen H., Koerten H.K., Spaan W.J.M. (1996). The Production of Recombinant Infectious DI-Particles of a Murine Coronavirus in the Absence of Helper Virus. Virology.

[7] Nieto-Torres J.L., DeDiego M.L., Álvarez E., Jiménez-Guardeño J.M., Regla-Nava J.A., Llorente M., Kremer L., Shuo S., Enjuanes L. (2011). Subcellular Location and Topology of Severe Acute Respiratory Syndrome Coronavirus Envelope Protein. Virology.

[8] Malik Y.A. (2020). Properties of Coronavirus and SARS-CoV-2. Malays. J. Pathol..

[9] Surya W., Li Y., Verdià-Bàguena C., Aguilella V.M., Torres J. (2015). MERS Coronavirus Envelope Protein Has a Single Transmembrane Domain That Forms Pentameric Ion Channels. Virus Res..

[10] Madan V., García M.D.J., Sanz M.A., Carrasco L. (2005). Viroporin Activity of Murine Hepatitis Virus E Protein. FEBS Lett..

[11] Ye Y., Hogue B.G. (2007). Role of the Coronavirus E Viroporin Protein Transmembrane Domain in Virus Assembly. J. Virol..

[12] Mandala V.S., McKay M.J., Shcherbakov A.A., Dregni A.J., Kolocouris A., Hong M. (2020). Structure and Drug Binding of the SARS-CoV-2 Envelope Protein Transmembrane Domain in Lipid Bilayers. Nat. Struct. Mol. Biol..

[13] Zhou S., Lv P., Li M., Chen Z., Xin H., Reilly S., Zhang X. (2023). SARS-CoV-2 E Protein: Pathogenesis and Potential Therapeutic Development. Biomed. Pharmacother..

[14] Fiore C., Eisenhut M., Krausse R., Ragazzi E., Pellati D., Armanini D., Bielenberg J. (2008). Antiviral Effects of Glycyrrhiza Species. Phytother. Res..

[15] Sun Z.-G., Zhao T.-T., Lu N., Yang Y.-A., Zhu H.-L. (2019). Research Progress of Glycyrrhizic Acid on Antiviral Activity. Mini Rev. Med. Chem..

[16] Pompei R., Pani A., Flore O., Marcialis M.A., Loddo B. (1980). Antiviral Activity of Glycyrrhizic Acid. Experientia.

[17] Hoever G., Baltina L., Michaelis M., Kondratenko R., Baltina L., Tolstikov G.A., Doerr H.W., Cinatl J. (2005). Antiviral Activity of Glycyrrhizic Acid Derivatives against SARS-Coronavirus. J. Med. Chem..

[18] Chrzanowski J., Chrzanowska A., Graboń W. (2021). Glycyrrhizin: An Old Weapon against a Novel Coronavirus. Phyther. Res..

[19] Bailly C., Vergoten G. (2020). Glycyrrhizin: An Alternative Drug for the Treatment of COVID-19 Infection and the Associated Respiratory Syndrome?. Pharmacol. Ther..

[20] Fomenko V.V., Rudometova N.B., Yarovaya O.I., Rogachev A.D., Fando A.A., Zaykovskaya A.V., Komarova N.I., Shcherbakov D.N., Pyankov O.V., Pokrovsky A.G. (2022). Synthesis and In Vitro Study of Antiviral Activity of Glycyrrhizin Nicotinate Derivatives against HIV-1 Pseudoviruses and SARS-CoV-2 Viruses. Molecules.

[21] Kang H., Lieberman P.M. (2011). Mechanism of Glycyrrhizic Acid Inhibition of Kaposi’s Sarcoma-Associated Herpesvirus: Disruption of CTCF-Cohesin-Mediated RNA Polymerase II Pausing and Sister Chromatid Cohesion. J. Virol..

[22] Sekizawa T., Yanagi K., Itoyama Y. (2001). Glycyrrhizin Increases Survival of Mice with Herpes Simplex Encephalitis. Acta Virol..

[23] Baba M., Shigeta S. (1987). Antiviral Activity of Glycyrrhizin against Varicella-Zoster Virus in Vitro. Antiviral Res..

[24] Lin J.C. (2003). Mechanism of Action of Glycyrrhizic Acid in Inhibition of Epstein-Barr Virus Replication in Vitro. Antiviral Res..

[25] Duan E., Wang D., Fang L., Ma J., Luo J., Chen H., Li K., Xiao S. (2015). Suppression of Porcine Reproductive and Respiratory Syndrome Virus Proliferation by Glycyrrhizin. Antiviral Res..

[26] Harada S. (2005). The Broad Anti-Viral Agent Glycyrrhizin Directly Modulates the Fluidity of Plasma Membrane and HIV-1 Envelope. Biochem. J..

[27] Crance J.M., Lévêque F., Biziagos E., van Cuyck-Gandré H., Jouan A., Deloince R. (1994). Studies on Mechanism of Action of Glycyrrhizin against Hepatitis A Virus Replication in Vitro. Antiviral Res..

[28] Sui X., Yin J., Ren X. (2010). Antiviral Effect of Diammonium Glycyrrhizinate and Lithium Chloride on Cell Infection by Pseudorabies Herpesvirus. Antiviral Res..

[29] Schröfelbauer B., Raffetseder J., Hauner M., Wolkerstorfer A., Ernst W., Szolar O.H.J. (2009). Glycyrrhizin, the Main Active Compound in Liquorice, Attenuates pro-Inflammatory Responses by Interfering with Membrane-Dependent Receptor Signalling. Biochem. J..

[30] Matsumoto Y., Matsuura T., Aoyagi H., Matsuda M., Hmwe S.S., Date T., Watanabe N., Watashi K., Suzuki R., Ichinose S. (2013). Antiviral Activity of Glycyrrhizin against Hepatitis C Virus In Vitro. PLoS ONE.

[31] Selyutina O.Y., Polyakov N.E., Korneev D.V., Zaitsev B.N. (2016). Influence of Glycyrrhizin on Permeability and Elasticity of Cell Membrane: Perspectives for Drugs Delivery. Drug Deliv..

[32] Selyutina O.Y., Polyakov N.E. (2019). Glycyrrhizic Acid as a Multifunctional Drug Carrier—From Physicochemical Properties to Biomedical Applications: A Modern Insight on the Ancient Drug. Int. J. Pharm..

[33] Glazachev Y.I., Schlotgauer A.A., Timoshnikov V.A., Kononova P.A., Selyutina O.Y., Shelepova E.A., Zelikman M.V., Khvostov M.V., Polyakov N.E. (2020). Effect of Glycyrrhizic Acid and Arabinogalactan on the Membrane Potential of Rat Thymocytes Studied by Potential-Sensitive Fluorescent Probe. J. Membr. Biol..

[34] Selyutina O.Y., Shelepova E.A., Paramonova E.D., Kichigina L.A., Khalikov S.S., Polyakov N.E. (2020). Glycyrrhizin-Induced Changes in Phospholipid Dynamics Studied by 1H NMR and MD Simulation. Arch. Biochem. Biophys..

[35] Selyutina O.Y., Apanasenko I.E., Kim A.V., Shelepova E.A., Khalikov S.S., Polyakov N.E. (2016). Spectroscopic and Molecular Dynamics Characterization of Glycyrrhizin Membrane-Modifying Activity. Colloids Surf. B Biointerfaces.

[36] Chernyshev A. (2020). Pharmaceutical Targeting the Envelope Protein of SARS-CoV-2: The Screening for Inhibitors in Approved Drugs. ChemRxiv.

[37] Pervushin K., Tan E., Parthasarathy K., Lin X., Jiang F.L., Yu D., Vararattanavech A., Tuck W.S., Ding X.L., Torres J. (2009). Structure and Inhibition of the SARS Coronavirus Envelope Protein Ion Channel. PLOS Pathog..

[38] Struppe J., Whiles J.A., Void R.R. (2000). Acidic Phospholipid Bicelles: A Versatile Model Membrane System. Biophys. J..

[39] Piai A., Fu Q., Dev J., Chou J.J. (2017). Optimal Bicelle q for Solution NMR Studies of Protein Transmembrane Partition. Chemistry.

[40] Zeng C.-X., Hu Q. (2008). Determination of the Polyacid Dissociation Constants of Glycyrrhizic Acid. Indian J. Chem..

[41] Lazaridis T., Mallik B., Chen Y. (2005). Implicit Solvent Simulations of DPC Micelle Formation. J. Phys. Chem. B.

[42] Leftin A., Molugu T.R., Job C., Beyer K., Brown M.F. (2014). Area per Lipid and Cholesterol Interactions in Membranes from Separated Local-Field 13C NMR Spectroscopy. Biophys. J..

[43] Björnerås J., Nilsson M., Mäler L. (2015). Analysing DHPC/DMPC Bicelles by Diffusion NMR and Multivariate Decomposition. Biochim. Biophys. Acta Biomembr..

[44] Salditt T., Li C., Spaar A. (2006). Structure of Antimicrobial Peptides and Lipid Membranes Probed by Interface-Sensitive X-Ray Scattering. Biochim. Biophys. Acta Biomembr..

[45] Chaturvedula V.S.P., Yu O., GuoHong M. (2014). NMR Analysis and Hydrolysis Studies of Glycyrrhizic Acid, a Major Constituent of Glycyrrhia Glabra. Eur. Chem. Bull..

[46] Panahi A., Brooks C.L. (2015). Membrane Environment Modulates the PKa Values of Transmembrane Helices. J. Phys. Chem. B.

[47] Selyutina O.Y., Kononova P.A., Polyakov N.E. (2020). Experimental and Theoretical Study of Emodin Interaction with Phospholipid Bilayer and Linoleic Acid. Appl. Magn. Reson..

[48] Thurlkill R.L., Grimsley G.R., Scholtz J.M., Pace C.N. (2006). Hydrogen Bonding Markedly Reduces the PK of Buried Carboxyl Groups in Proteins. J. Mol. Biol..

